# The impact of COVID-19 in plastic surgery departments: a comparative retrospective study in a COVID-19 and in a non-COVID-19 hospital

**DOI:** 10.1007/s00238-020-01725-w

**Published:** 2020-08-26

**Authors:** Francesca Romana Grippaudo, Emilia Migliano, Ugo Redi, Gianmarco Turriziani, Davide Marino, Giuseppe D’Ermo, Diego Ribuffo

**Affiliations:** 1 Department of Surgery “P. Valdoni”, Sapienza University of Rome, Rome, Italy; 2 Plastic and Regenerative Surgery Dept., San Gallicano Dermatological Institute IRCCS, Rome, Italy; 3 Unit of Plastic and Reconstructive Surgery, Policlinico Umberto I, Department of Surgery “P. Valdoni”, Sapienza University of Rome, Rome, Italy

**Keywords:** COVID-19, Plastic surgery, Skin cancer, SARS, Pandemic

## Abstract

**Background:**

COVID-19 is a new human-infecting coronavirus for which the World Health Organization declared a global pandemic. The first Italian cases occurred in February 2020: since then, there has been an exponential increase in new cases, hospitalizations and intensive care assistance demand. This new and sudden scenario led to a forced National Health System reorganization and review of welfare priorities. The aim of this study is to evaluate the effects of this pandemic on ordinary activities in two plastic surgery divisions in Rome, hosted in a COVID-19 and a non-COVID-19 hospital.

**Methods:**

The data of this comparative retrospective study was collected between 9 March and 9 April 2019 and the same period of 2020 from two plastic surgery units, one in a COVID-19 hospital and second in a non-COVID-19 hospital in Rome, Italy. The 2019–2020 data of the two hospitals was compared regarding the number of surgeries, post-operative dressings and first consultations performed.

**Results:**

Both units sustained a decrease in workload due to lockdown effects. Statistically significant differences for day surgery procedures (*p* value = 0.0047) and first consultations (*p* value < 0.0001) were found between the COVID-19 and non-COVID-19 institutes, with a drastic trend limiting non-urgent access to COVID-19 hospitals.

**Conclusions:**

The long-term effects of healthcare reshuffling in the “COVID-19 era” imply a delay in the diagnosis and treatment of skin cancer and cancellation of many reconstructive procedures. These findings pose a question on the future consequences of a long-term limitation in plastic surgery healthcare.

Level of evidence: Level III, risk/prognostic study.

## Introduction

COVID-19 is a new human-infecting *Betacoronavirus*, first reported in Wuhan (China) in December 2019 and rapidly spreading to all continents, causing a pandemic and a public health emergency. This virus is highly contagious with a human-to-human transmission and may present a benign course showing flu-like symptomatology (malaise, fever, cough) or a serious health hazard with severe acute respiratory syndrome (SARS), acute cardiac injury and acute kidney injury [1, 2], among other systemic effects described.

Contact frequency among individuals is known as one of the major elements affecting the spread of the disease. Liu et al. estimate the basic reproduction number (*R*_0_) of COVID-19, a mathematical term that indicates how contagious a disease is, indicating the average number of people who will catch COVID-19 from one single infected patient as 3.8 [3]. Transmission from asymptomatic carriers has been demonstrated. Italy has been highly affected by this pandemic since February 2020 [4], with 173, 730 confirmed cases and 22, 586 deaths according to the data of ‘Istituto Superiore di Sanità’ on 22 April 2020 [5]. In Rome, the number of patients infected to date is 4257. The Italian National Health System is currently facing a challenge due to the high demand for intensive care assistance needed by 9–11% of COVID-19 patients [6] and the lack of beds in intensive care units. Therefore, remarkable efforts are spent to provide an efficacious reaction to the emergency, reorganizing the beds within the public health system hospitals to create new beds for COVID-19 patients. Italian hospitals have started to reduce elective activities to receive the high number of infected patients [7], and in an endeavour to preserve normal activities, ‘COVID-19’ and ‘non-COVID-19’ hospitals were identified in the NHS hospital network.

It only makes sense that today’s focus is exclusively on the SARS-CoV-2, and the hospitals are primarily acting to defeat it. The coronavirus has deleted everything that can be felt superfluous and/or unnecessary. After the Prime Ministerial Decree 09 March 2020 [8], the two leading Italian plastic surgery organizations, SICPRE (Italian Society of Plastic, Reconstructive and Aesthetic Surgery) [9] and AICPE [10] (Association of Aesthetic Plastic Surgery) provided recommendations to postpone any routine elective plastic surgery, with the exception of cancer or emergencies.

Most of the Italian plastic surgery wards faced a reduction in beds and theatres to enable hospitals to free up healthcare staff to provide medical care for patients in other areas, given the need for a change in work organization to comply with limited outpatient clinic activities and reduced theatre availability for all hospitalization typologies and to cope with new pre-hospitalization modalities to screen up COVID-19 positive patients among the ones scheduled for surgery.

The aim of this comparative retrospective study is to ascertain the effects of the COVID-19 pandemic on ordinary activities in two plastic surgery division in Rome, Italy, one in a COVID-19 hospital and the other in a non-COVID-19 hospital.

## Materials and methods

This is a comparative retrospective study. Data was collected from two plastic surgery divisions in Rome, Italy, of which Policlinico Umberto I (PU1) was set as a COVID-19 hospital and San Gallicano (ISG) as a non-COVID-19 hospital. PU1 Plastic Surgery Department serves the Faculty of Medicine and Dentistry at Rome’s Sapienza University, Italy, with a staff consisting of six consultants and eleven trainees. In 2019, the in-hospital ward had ten beds and five weekly theatres treating 500 patients; the day surgery ward had three beds and five weekly theatres treating 750 patients; 1610 outpatient clinic surgery operations were performed and 10. 500 outpatient consultations were carried out, of which 3.360 were referrals and 7.140 were dressing changes.

ISG Plastic Surgery Department is located in a Roman IRCSS, a biomedical institution of relevant national interest, which drives clinical assistance in strong relation to research activities. The staff is made up of nine consultants and one trainee. In 2019, the in-hospital ward had seven beds and five weekly theatres treating 341 patients; the day surgery ward had four beds and five weekly theatres treating 981 patients; 1.655 ambulatory surgery operations were performed and 9.151 outpatients received a consultation, of which 3.635 were referrals and 5.516 were dressing changes.

The study analysed the data collected between 09 March (starting lockdown date in Italy) and 09 April 2020 and the same period of 2019. Outpatient, day surgery (DS) and in-patient (IP) medical charts were retrieved from both plastic surgery departments and the following data compared:

- The total number of patients treated in both hospitals and in each hospitalization regimen
- The pathologies operated on in-patients and day surgery regimens of care
- The total monthly number of theatre hours for DS patients and for in-patients
- The total monthly ward beds given to each unit in DS and in IP regimen of care
- The number of staff members on duty
- The pre-hospitalization screening tests and the admittance criteria to the plastic surgery department

Statistical analysis on retrieved data was conducted with the chi-squar test (significance level of *p* < 0,05) using SPSS statistics version 18 (SPSS Inc., IBM Company, Chicago, IL).

## Results

**Fig. 1:**
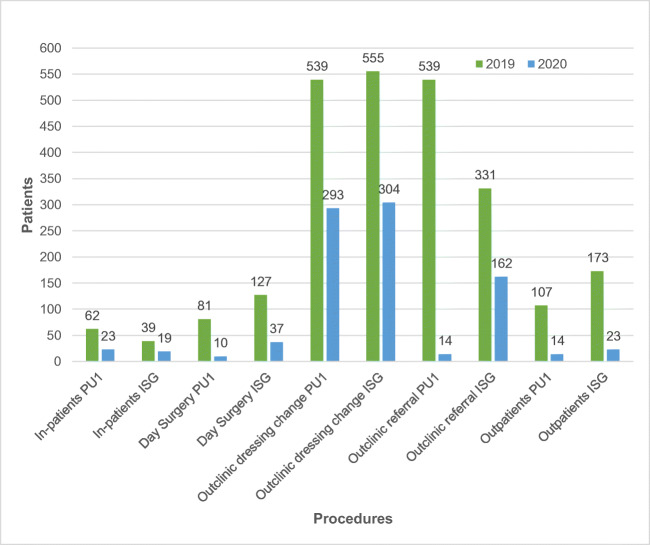
Total number of patients treated from 09 March to 09 April 2019 and the same period of 2020 as in-patient, day surgery, out-clinic setting (divided into dressing change and referral/first visit) and outpatient surgery in both departments. Data from PU1 shows a reduction in the number of in-patients by 62.9% from the outbreak, 87.6% for day surgery procedures and 86.9%. for outpatient surgery. The percentage of the total number of outpatient dressing change decreased by 43.4% from the outbreak, whilst the percentage of the first consultations dropped by about 97.4%. Data from ISG shows a reduction of in-patient number by 51.2% from the outbreak, 70.9% for day surgery procedures and 86.7% for outpatient surgery. The percentage of dressing change decreased by 45.2%, whilst the referrals diminished by 51% from the outbreak

Both units sustained a decrease in workload due to the lockdown effects (Fig. 1).

Routine follow-up visits were suspended and replaced by phone calls where feasible, except for dressing change in recently discharged patients. Only patients referred as urgent by the general practitioner were scheduled for consultation.

PU1 in-patient ward capacity was reduced to 6 beds, to accomplish the 2-m social distance between beds; ISG in-patient ward capacity was reduced to 4, thus accommodating one patient only in an originally double-bed room.

Theatre availability was reduced as well, in accordance with the work volume. Outpatient clinic surgery was considerably reduced in both departments: 90% in PU1 and 80% in ISG compared with the same period in 2019. In both hospitals, only melanoma was treated, excluding basal cell carcinoma and squamous cell carcinoma.

In both units, visitors for day surgery patients were not allowed; for in-patients, only one visitor per room was allowed, after a ThermoScan check negative for fever. All patients and visitors were required to wear a surgical mask during their permanence on the hospital grounds.

Consultant staff shift remained unchanged in PU1 to help in COVID-19 patients care, and daily resident number was reduced to two; while in ISG a restricted staff policy was adopted to limit exposures, limiting the staff on duty to two surgeons each day and resident on duty only when surgery was scheduled.

From 4 March 4 2020 onwards, all patients requiring admission to both plastic surgery departments were screened 24 h prior to admission, by means of a telephone interview by a doctor from each unit, to triage a possible COVID-19 infection that would contraindicate hospital admission and require treatment in the appropriate setting (Table 1).

**Table 1:**
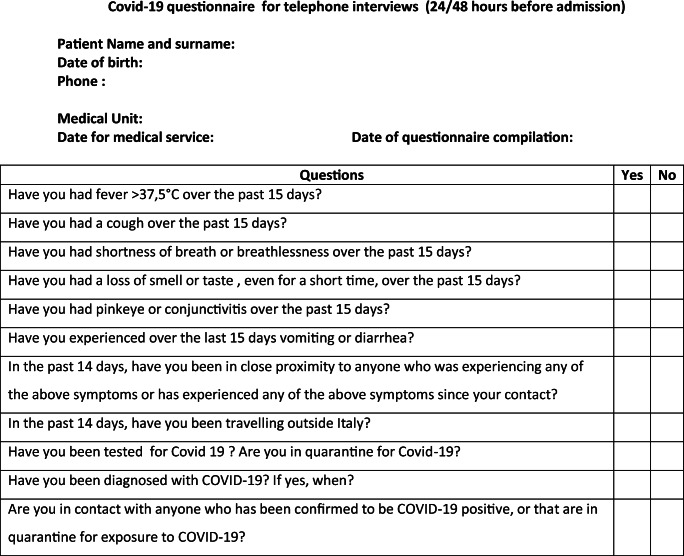
Covid-19 questionnaire for telephone interviews (24/48 h before admission)

All patients in both hospitals also had to complete a preoperative health screening prior to admission, including one negative COVID-19 test using the reverse transcription polymerase chain reaction on specimens from both upper respiratory tracts (nose and oropharyngeal samples), taken at least 48 h before scheduled surgery. All non-oncologic surgery was curtailed in both hospitals.

When compared with the same period of 2019, 2020 witnessed a percentage decrease with regard to in-patient and outpatient procedures in both hospitals.

**Fig. 2:**
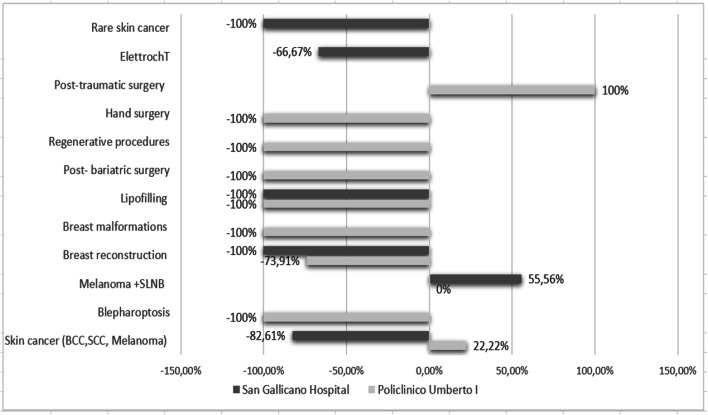
Percentage change in in-patient procedures in ISG and PU1, during the period 9 March–9 April 2020 and the period 9 March–9 April 2019. Melanoma + sentinel lymph node biopsy (SLNB) is the only increased surgery for ISG, whereas skin cancer and post-traumatic surgeries are the increased procedures for PU1: post-traumatic surgery is doubled (+ 100%). In PU1, melanoma + SLNB has not changed (0%). All other surgeries decreased in both hospitals (− 100% means that the procedure has not been performed)

In detail, PU1 faced a total in-patient surgery decrease of 62.90%, while at ISG it amounted to 51.28%. Figure 2 shows the specific variation by type of in-patient surgery and highlights the reduction in non-urgent procedures such as lipofilling, post-bariatric surgery or periorbital surgery and the increase in surgical oncology and trauma surgery.

Ambulatory surgery decreased by 90% at PU1 and by 80% at ISG when compared with the same period in 2019.

Day surgery procedures decreased by 87.65% at PU1 and by 70.87% at ISG.

The average number of in-patient hospitalization days between 2019 and 2020 remained almost unchanged for PU1 (from 6.4 to 6.8 days) and for ISG (from 2.8 to 2.5 days).

Overall, there is a clear decrease in welfare procedures in both hospitals, with statistically significant differences at chi-square test between the two institutes for day surgery procedures and first consultations (Table 2)**.**

**Table 2:** Chi-square analysis among the medical activities of the two units, showing a statistically significant reduction of referrals and day surgery procedures only in PU1, when compared with ISG. No significant differences were detected in the remaining activities

Differences between the decreases in percentage in PU1 and ISG procedures	*p* value
Day surgery procedures	0.0047*
In-patient procedures	0.2486
Outpatient procedures	0.9597
Overall surgical procedures	0.1877
Wound dressings	0.6
Referrals	0.0001*

*Statistically significant

## Discussion

The primary objective of this study was to ascertain if there was a qualitative and quantitative modification in the activities of plastic surgery departments caused by the COVID-19 pandemic. The secondary end-point was to ascertain whether the nature of COVID-19 hospital or non-COVID-19 hospital hosting plastic surgery division had any influence on such result.

The pandemic-based guidelines of state authorities in many countries stipulate that all elective procedures that could be safely delayed must be cancelled [11, 12] until the end of the pandemic, limiting the number of exposures for healthcare workers and reducing nosocomial transmission [13].

Despite the national government decree, plastic surgery activities show different managements depending on the COVID-19 or non-COVID-19 nature of the host hospital.

After this work, it is possible to ascertain that both plastic surgery departments enrolled in this study are facing an overall decrease in activities, with a substantial cut in plastic surgery cares, which normally include a wide spectrum of diseases.

Study data shows the effects of the consequence of cancellation of plastic surgery elective surgeries in both hospitals during the lockdown, when the operating theatre was available only for such urgent procedures as melanoma or melanoma and SLNB removal or for post-traumatic reconstruction in PU1. These non-delayable procedures were unaffected in both hospitals, and this fact caused a relative increase in trauma surgeries and oncological figures compared with 2019. That is not due to a surge of patient population affected by these pathologies but reflects the drop in elective procedures.

Due to the reduced availability of operating theatres and the restricted hospital policy admission criteria, the limited numbers of staff on duty were sufficient in both hospital to cover all the activities.

The data qualitative analysis showed a similar decrease in both units about in-patient and day surgery cases and a statistically significant difference in workload between the units concerning the outpatient surgery and the referrals.

This reduction in health services had a higher impact in PU1, where some of the anaesthetist staff and intensive care beds were recruited for COVID-19 patients. Another reason for this difference is that patients requiring referrals or outpatient surgery are more apt to avoid COVID-19 hospital for fear of nosocomial transmission and, accordingly, ISG endured a lesser drop in these activities.

Procedures that are delayed until elective surgery because deemed safe include basal cell carcinoma removal, secondary breast reconstruction, post-bariatric surgery, regenerative medicine, hand surgery and electrochemotherapy for the treatment of cutaneous and non-cutaneous cancer.

Fuertes described the impact of COVID-19 pandemic in Spanish plastic surgery units on twelve plastic surgery unit across Spain, investigating on different effects of the pandemic: team members schedule reduction, variation in type and number of surgical procedure, etc. [14]

Fuertes results are comparable to ours in respect to the drastic reduction in overall surgical procedures (in-patient and outpatient) and consults, with a prevalence of oncologic case and a postponement of elective surgical activity. In this report, one hospital only, geographically located in a mildly affected pandemic area, declared to have maintained its usual activities. Staff policy reductions were applied also in Spain, with effects on increase of shifts numbers per consultants.

At present, 6 weeks after the lockdown began, there is no scheduled date yet to plan the return to full activities in both COVID-19 and non-COVID-19 hospitals, with the next national government guidelines expected on 19 May. As a result, some patients could be damaged because of an undetected worsening of a long-standing lesion while in waiting list for planned elective surgery. Other surgical specialties are facing the same problem, due to the restriction in elective surgery procedures [15, 16]. Emergency surgery addresses a broad spectrum of diseases of traumatic genesis or acute illness that need surgical treatment [17]. Conversely, elective surgery does not mean optional surgery but identifies a procedure assigned to a pathology that is not life-threatening in the immediate term and yet can seriously harm the patient if postponed for a long time [18]. Most of the procedures delayed by the plastic surgery units in this study are included in this definition. Brücher et al. in a comprehensive article on pandemic surgery guidance described three surgical response phases depending on the epidemiological situation of COVID-19: phase 1 with only few COVID-19 patients, infection rate not in rapid increase and good availability of intensive care unit (ICU) beds and ventilators; phase 2 with many COVID-19 patients and limited capacity of hospital and ICU resources; and phase 3 when all hospital resources are diverted to COVID-19 healthcare and only life-saving operations are performed [19]. When this manuscript was drawn up, Rome was in phase 2, although all of Italy was declared a red zone with similar restrictions in access to healthcare. Therefore, a possible bias of this study is that it can be better compared only in regions in the same phase of the pandemic, since regions in which the pandemic has had the highest numbers will be much worst and, conversely, in regions with less COVID-19 patients the figures will be better. Further studies are needed to evaluate the consequences of COVID-19 induced healthcare limitations in this class of patients with non-urgent pathologies.

## Conclusions

This is a preliminary study that evaluates the current situation in Italian plastic surgery units amid the COVID-19 outbreak. The decrease in procedures has relevant economic implications not to be underestimated. We are now working on guidelines in the event of similar future scenarios since, to date, we are not able to predict the foreseeable events.
